# Increased Prevalence of Alloimmunization in Sickle Cell Disease? Should We Restore Blood Donation in French Guiana?

**DOI:** 10.3389/fmed.2021.681549

**Published:** 2021-06-11

**Authors:** Salomé Conrath, Vincent Vantilcke, Mickael Parisot, Françoise Maire, Pierre Selles, Narcisse Elenga

**Affiliations:** ^1^Sickle Cell Disease Center, Andrée Rosemon Regional Hospital, Cayenne, French Guiana; ^2^Etablissement Français du Sang, Andrée Rosemon Regional Hospital, Cayenne, French Guiana; ^3^Pediatric Medicine and Surgery, Andrée Rosemon Regional Hospital, Cayenne, French Guiana

**Keywords:** sickle cell disease, immunohematological issues, transfusion, alloimmunization, delayed hemolytic transfusion reaction

## Abstract

Patients with sickle cell disease often undergo frequent blood transfusions. This increases their exposure to red blood cell alloantigens of donor units, thus making it more likely that they produce alloantibodies. This cross-sectional study aimed to describe the prevalence of allo-immunization in patients with sickle cell disease who were monitored at Cayenne Hospital in 2016. Of the 451 patients recruited during the study period, 238 (52.8%) were female. There were 262 (58.1%) homozygous sickle cell and 151 (33.5%) compound heterozygous sickle cell patients. The median age of the participants was 23.09 years (range, 0.5–68). We noted different red blood cell extended phenotypes: -in the Duffy system, the Fy^a^- Fy^b^–profile was found in 299 patients (66%);—for the Kidd system, the most represented profile was Jka+ Jkb-, with 213 patients (47%). The Jka antigen was present in 355 patients;—in the MNS system, the S-s+ profile was found in 297 patients (66%);—the Lea antigen of the Lewis system was absent in 319 patients. The most frequent Rh phenotype in our patients was D+ C- E- c+ e+ K-, representing 51% of the patients. A total of 6,834 transfused packed red blood cell units were recorded. Sixty-eight patients (23%; 95% confidence interval, 20–25%) had detectable RBC alloantibodies. In multivariate logistic regression, only the mean number of single transfusions was statistically higher for the alloimmunized patients (*p* < 0.04). Thirteen (19%) of the patients with alloimmunization developed a delayed hemolytic transfusion reaction, thus representing 4.4% of the total number of transfused patients. Whether differences between donors from France vs. recipients from French Guiana could explain this high prevalence of alloimmunization to be examined. In conclusion, careful transfusion strategies for patients with RBC alloantibodies should allow further reduction of the rate of alloimmunization.

## Introduction

Red blood cell (RBC) transfusions are often used to treat acute complications of sickle cell disease (SCD). The purpose of RBC transfusion is to increase oxygen distribution to the tissues and/or to replace the rigid sickle-shaped RBCs with healthy deformable RBCs (1). Transfusion can also be part of a regular long-term transfusion program aimed at the prevention of SCD complications. Seventeen percent of SC patients, 45% of S/β-thalassemia patients, and more than 60% of SS homozygous patients undergo at least one transfusion before the age of 18 years. It is estimated that 90% of adults with sickle cell disease will receive at least one transfusion in their lifetime (2). Many patients with SCD, however, undergo numerous transfusions over the course of their lives, thereby greatly increasing their exposure to RBC alloantigens of donor units. These patients are, therefore, more likely to produce alloantibodies. RBC alloimmunization can cause adverse effects, resulting in delayed hemolytic transfusion reactions (DHTRs) and limit the further safe transfusion. The incidence of alloimmunization in SCD has been reported to be 20–50% (3, 4). Recognition of RBC antigens, proteins and membrane glycoproteins by the recipient immune system leads to generation of antibodies and memory cells (5, 6). Alloimmunization is a frequent occurrence due to antigen disparities between patients and donors. Its severity varies according to the number of transfusions received and according to phenotypic differences between the recipients (who are often of African descent) and blood donors (who tend to be of European ancestry) (7, 8). Exact matching for ABO, Rhesus, Kell, Kidd, and Fy^a^ blood group antigens, and extending this match whenever possible, in patients who have not yet developed immunity is an effective strategy for reducing alloimmunization to RBC antigens in patients with sickle cell disease. French Guiana is an overseas French territory located in South America. It is bounded by Brazil to the south and east, Suriname to the west, and the Atlantic Ocean to the Northeast. The incidence of SCD in French Guiana is ~0.41% (9). Following four deaths related to Chagas disease, a serological survey conducted in 2004 revealed a prevalence of Chagas disease of 0.5% among blood donors in French Guiana. In April 2005, blood drives in French Guiana were discontinued due to concerns of the safety of blood products (10). Other barriers to blood donation in French Guiana are a number of contagious infectious diseases with high risk for transfusion transmission, such as arboviruses (emergent and circulating), leptospirosis, Q fever, malaria, human immunodeficiency virus (HIV), and human T-cell leukemia virus (HTLV) infections. Two-thirds of French Guiana's supplies of packed RBCs comes from mainland France, and one-third is obtained from the Guadeloupian French Blood Establishment. The packed RBC units are transported by air to the Guianese French Blood Establishment. The latter facility only ensures their delivery to the hospitals where the transfusion is to be provided. This study aimed to describe the prevalence of allo-immunization in patients with SCD, since the transition away from blood donation in French Guiana.

## Materials and Methods

### Study Design

This was a cross-sectional study designed to describe the following immunohematological characteristics of sickle cell patients followed in our center: the number of transfusion episodes, number of units of transfused blood, indication for transfusion, age at first transfusion, history of pregnancy if necessary, blood group ABO, D and the extended phenotypes, the possible type of alloantibodies, the rate of alloimmunization, and types of red blood cell alloantibodies in patients with SCD who were being monitored at the sickle cell center of Cayenne Hospital in 2016. In our center, all patients are systematically tested for autoantibodies during the annual check-up. The study population was part of a pre-existing cohort titled: Improving the quality of management of SCD in French Guiana: “Epidemiology of predictive factors of acute clinical events,” that enrolled ~1,000 patients.

### Transfusion Procedures

In our center, as soon as SCD is diagnosed, ABO RH-K blood grouping and extended phenotyping are performed. The latter is sometimes made difficult when the patient has already been transfused, especially for transfusions performed abroad or in case of loss of records. The determination of the phenotype by molecular biology is then necessary. Before each transfusion, a test for irregular antibodies and a cross-matching test are systematically performed in the laboratory. Another cross-matching test is performed at the patient's bed. A control of irregular antibodies is planned 3–6 weeks after each transfusion.

All patients undergo the same anti-erythrocyte antibody screening protocol. This screening is performed using a three-cell panel and a gel test technique (indirect antiglobulin test). If antibodies are detected, a panel of 15 cells is used for identification, supplemented if necessary by a panel of 15 red blood cell cells treated with an enzyme (papain and/or trypsin). The distinction between alloantibodies and autoantibodies is based on the serological test, the phenotyping of red blood cells and, sometimes, the genotyping of red blood cells (in this case the sample is sent to a laboratory in France). Anti-M (anti-MNS1), anti-Lea (anti-LE1), anti-Leb (anti-LE2) and anti-Lex (anti-LE3) antibodies are considered as natural antibodies if they are present before any transfusion.

The transfusion protocol applied in our center, as in other French centers has been recently detailed in the paper by Clément et al. (11). Patients on a manual transfusion exchange program received 25 ml RBC/kg body weight every 4 weeks, preceded by phlebotomy if the hemoglobin level was >80 g/L. An HbS level of <30–40% was targeted depending on the indications. Since April 2012, manual exchange transfusion has been replaced by an automated exchange transfusion.

### Study Site

This study was conducted at the sickle cell center of Cayenne Hospital. Cayenne Hospital is a 510-bed general hospital center that serves as a referral and teaching hospital, and it has the only sickle cell center for children and adults (created in September 2014). The study took place between October 1 and December 31, 2016.

### Study Subjects

Consecutive patients with SCD (homozygous SS, compound heterozygous SC and S-β-thalassemia) attending the sickle cell center at Cayenne Hospital who were 6 months of age or older, with or without a history of blood transfusion, and who freely provided their consent (or whose parents provided consent) to participate were included in the study. All individuals who did not meet the inclusion criteria were excluded from the study. Since 1990, all patients receive leukoreduced, non-irradiated RBC units that are phenotypically matched for D, C/c, E/e, and K. In case of alloimmunization, patients are expected to receive erythrocyte units matched for the relevant antigens and for Fya, Fyb, Jka, Jkb, S and s, if more than two alloantibodies are identified (11).

### Data Collection

In the absence of a single computerized patient database, data from different sources (including the medical records from the French Guianese Blood Establishment, which monitored transfusions from 1995 to 2016) were collected in order to be as thorough as possible and to cross-check the information. The data were obtained directly from computerized hospital records or from paper records when computer data were not available. Records at the sickle cell center regarding the recruited SCD patients were reviewed in regard to their demographic characteristics and the transfusion history, the number of transfusions, the number of units of transfused blood, the date of the transfusions, the indication for transfusion, the age at the time of the first transfusion, and any history of pregnancy. The blood group ABO, D antigen, the extended phenotypes, and the possible type of alloantibodies were also recorded. All of these data were recorded in an anonymous Excel database. A delayed hemolytic transfusion reaction was suspected when a patient presented several days after RBC transfusion with the development or intensification of symptoms suggestive of a painful crisis, hemolysis, reticulocytopenia, and worsening anemia, reversing the effects of the previous RBC transfusion (12).

### Statistical Analysis

We used as primary outcome the prevalence of alloimmunization. This prevalence was calculated by dividing the number of alloimmunized patients by the total number of transfused patients. The alloimmunization rate was calculated by dividing the number of alloimmunized patients by the total number of RBC units received (before first alloimmunization). Statistical software packages (LibreOffice Calc version 5.1.6.2 and *Stata 12.0 Statistical Software: Release 12*. College Station, TX: StataCorp LP) were used for data management and analysis, respectively. For univariate analysis of possible associations between alloimmunization and gender, age at the time of the first transfusion, pregnancy history, the number of transfusions, the clinical and laboratory findings, the Chi-squared test or Fisher's exact test was used for discrete variables. Unconditional logistic regression analysis was used for the continuous variables with a non-Gaussian distribution. *P* < 0.05 was chosen as the threshold for statistical significance.

### Ethical Considerations

The patients, their parents, or authorized representatives provided written and informed consent to participate in this research. This study was approved by the hospital's ethics committee, and the database was declared to the Commission nationale de l'informatique et des libertés (Number 3Yj157849 3#).

## Results

**Figure 1:**
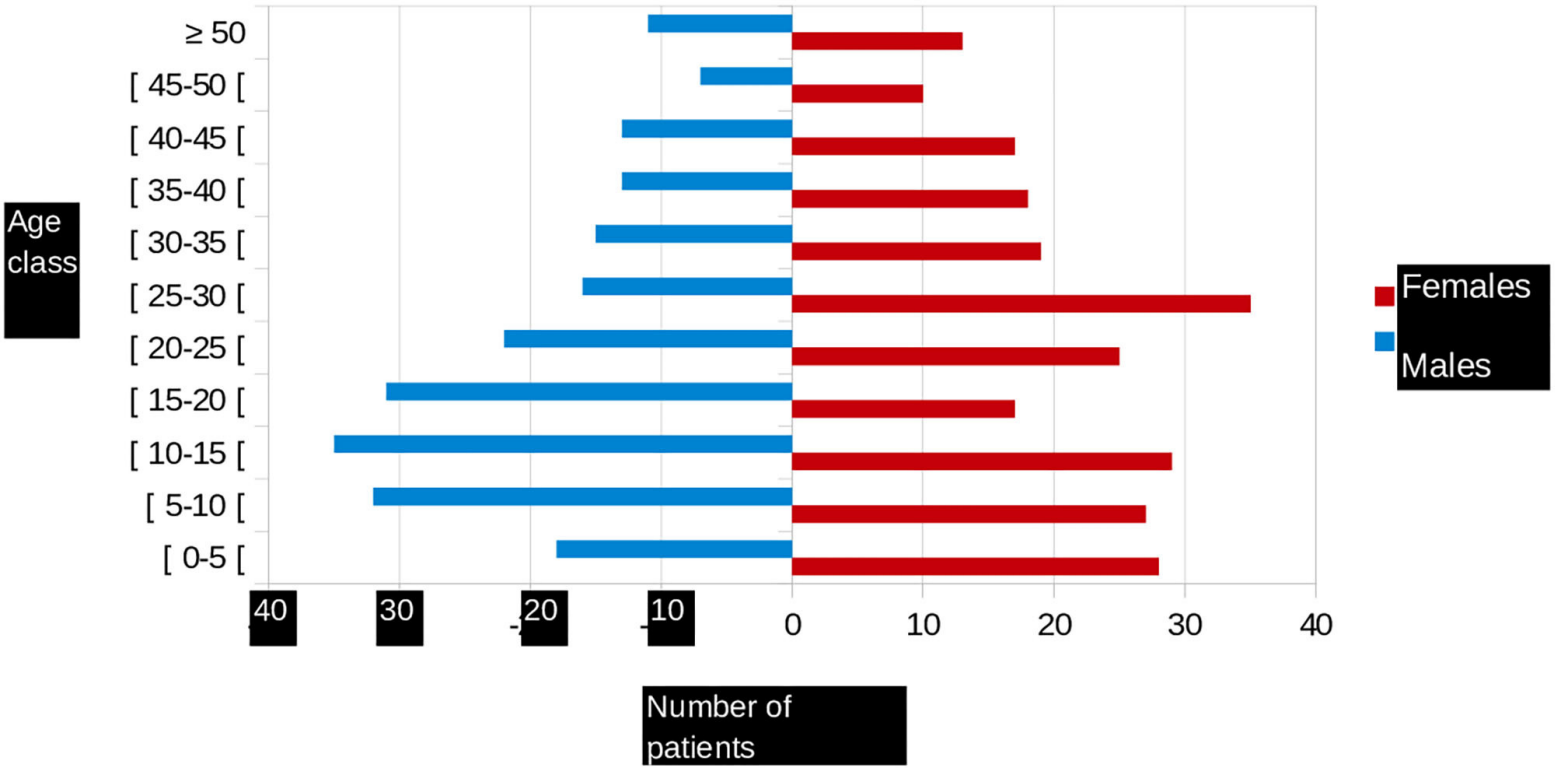
Age pyramid of patients followed in French Guiana.

Of the 451 patients recruited during the study period, 238 (52.8%) were female. There were 262 (58.1%) homozygous sickle cell and 151 (33.5%) compound heterozygous SC patients. The median age of the participants was 23.09 years (range, 0.5–68) (Figure 1). The most common blood group was O (51%), followed by group A (22%), and group B (17%). For 362 patients (80%), the RH/KEL group phenotype and an extended phenotype (Duffy, Kidd, MNS, and Lewis systems) were determined. Irrespective the extended phenotype, we noted different profiles, including:

1. in the Duffy system, the Fy^a^- Fy^b^- profile was found in 299 patients (66%);
2. for the Kidd system, the Jka+ Jkb- profile was the most common: 213 patients (47%). The Jka antigen was present in 355 patients;
3. in the MNS system, the S-s+ profile was found in 297 patients (66%);
4. the Lea antigen of the Lewis system was absent in 319 patients.

The most frequent Rh phenotype in our patients was D+ C- E- c+ e+ K-, representing 51% of all cases. Only 148 (33%) patients underwent molecular biology-based screening for a variant antigen or a rare phenotype. These are found in 40% of transfused sickle cell patients. Of the patients who were non-transfused or who had undergone few transfusions (<30 packed RBC transfusions), only 132 (33%) were assessed in this manner. Forty-two percent of the 103 patients who had undergone few blood transfusions were positive based on this molecular biology-based screening.

**Figure 2:**
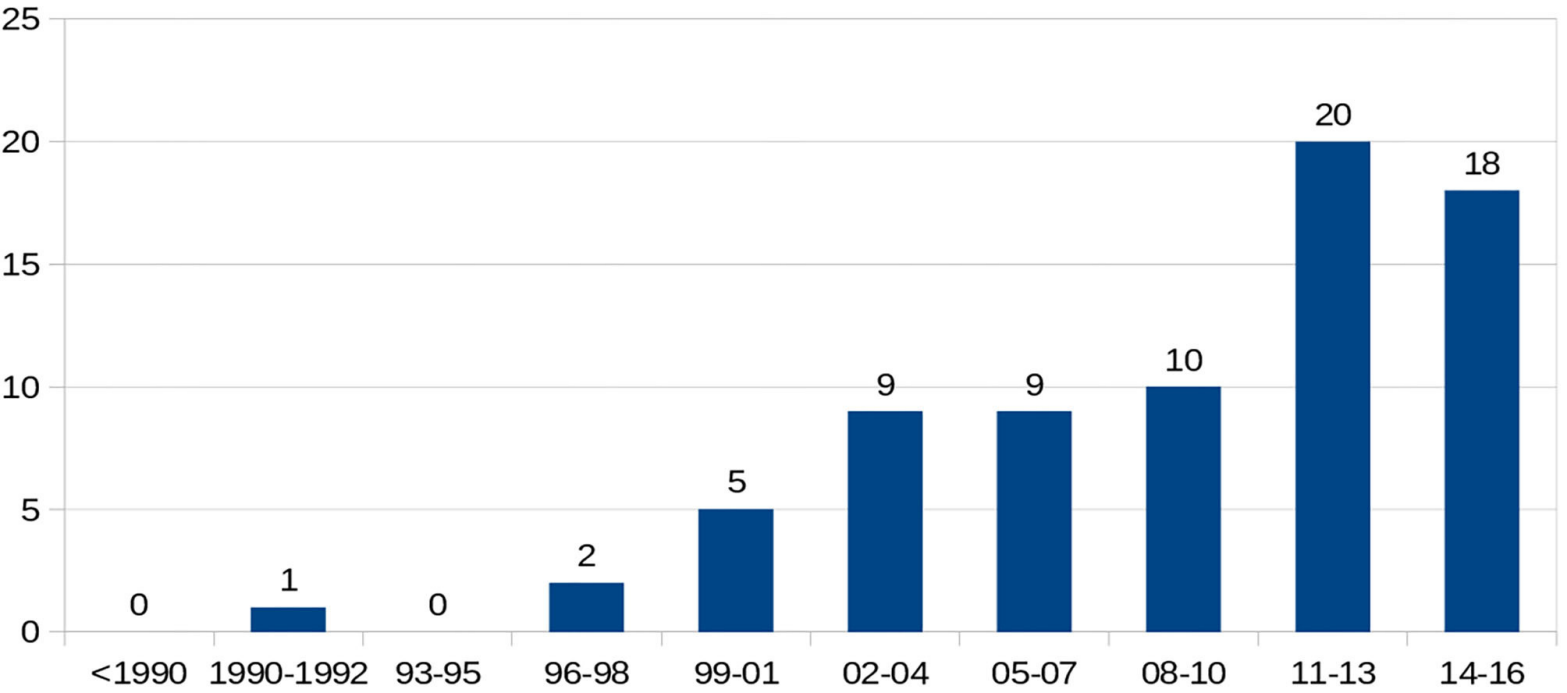
Temporal evolution of number of alloimmunization.

**Figure 3:**
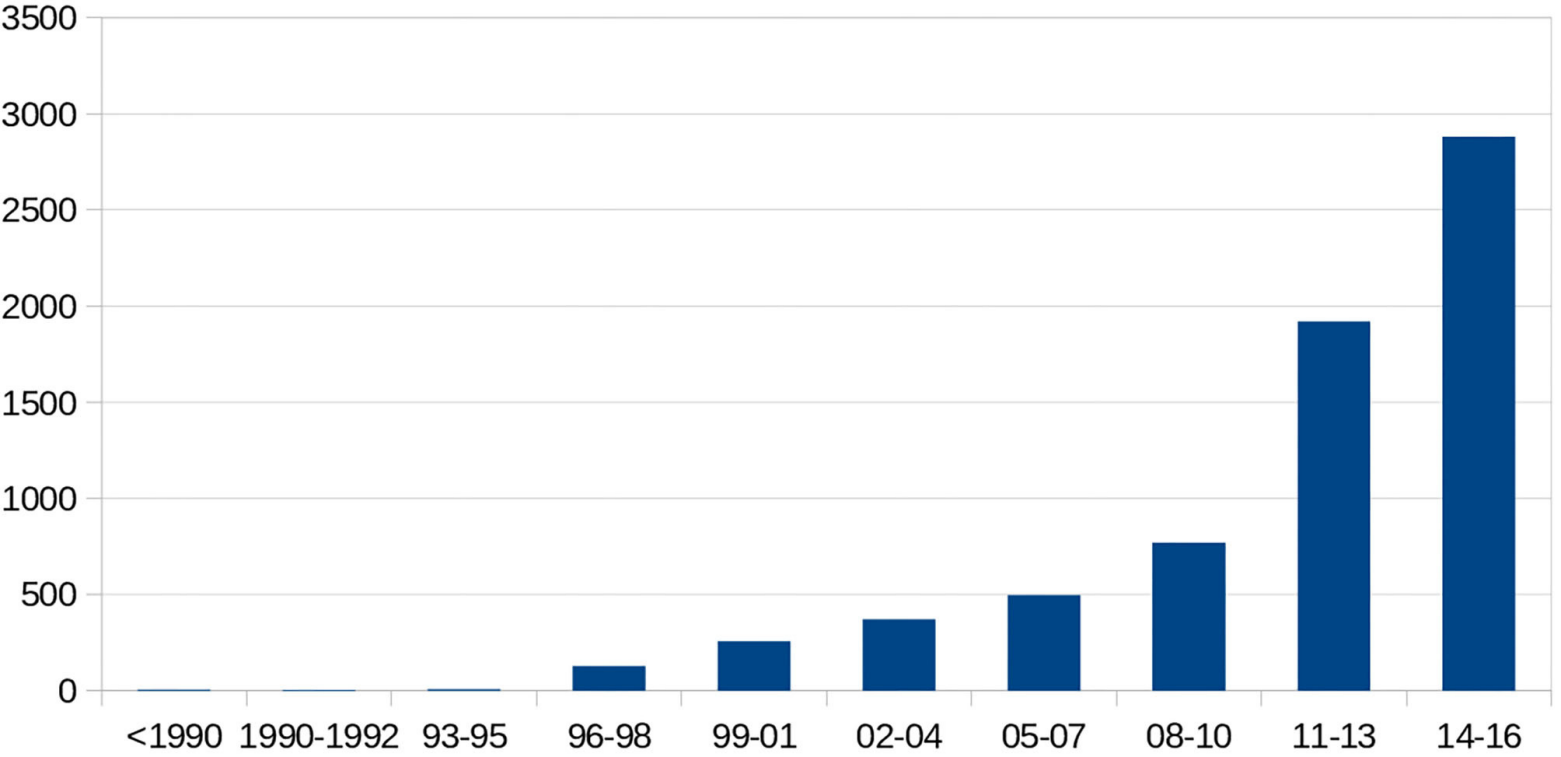
Temporal number of transfused packed red blood cells.

There were 298 (66%) sickle cell patients who had been transfused with at least one packed RBC transfusion, compared with 153 patients (34%) who had never been transfused.Twenty-one (4.7%) of the sickle cell patients had a history of at least one autoantibody, and 74 (16.4%) had a history of at least one alloantibody. The types of alloantibodies found in immunological reactions are detailed in Table 1. In total, 37 patients developed only one alloantibody, while 14 patients developed two different alloantibodies, including at least one alloantibody, while 23 other patients had at least three or more different alloantibodies, including at least one alloantibody. The number of alloimmunizations over time revealed an increase in these events, as shown in Figure 2. In parallel, the rate of RBC transfusions increased over time among the sickle cell patients, as shown in Figure 3.

**Table 1:** Specificities of the 134 RBC alloantibodies identified in 68 SCD patients in French Guiana.

**Blood group system**	**RBC alloantobody specificity**	**Number of alloantibodies respectively**	**Total *n* (%)**
Rhesus	C, Cw, D, E, e, RH20	6, 3, 7, 5, 2, 2	25 (19)
Kell	K1, K2, K6	3, 3, 2	8 (6)
Duffy	Fy1a, Fyb, Fy3	6, 1, 1	8 (6)
Kidd	Jya, Jyb	5, 7	12 (9)
MNS	M, S, MNS30	14, 13, 1	28 (21)
Lutheran	Lua	3-	3 (2)
Lewis	Lae, Leb, Le3ab	22, 13, 7	42 (31)
Non specific antibodies		8-	8 (6)

### Characteristics of the Transfused Patients

There were 298 sickle cell patients who had undergone at least one transfusion (51% aged under 18 years and 77% over 18 years of age). Twenty-six of them (8.7%) were transfused with only one packed RBC unit, 161 (54%) received fewer than 10 packed RBC units, and 137 (46%) 10 or more packed RBC units. The average number of packed RBC units received per transfused patient was 23, with a minimum of 1 and a maximum of 461 (standard deviation of 40.77). Fifty-nine (19.8%) of the transfused sickle cell patients had undergone exchange transfusions. Of these transfused patients, 68 (23%; 95% confidence interval, 20–25%) had detectable alloantibodies.

### Characteristics of the Transfused Packed RBC

A total of 6,834 packed *RBC* unit transfusions were recorded. No ABO incompatibility was found. Seventy-six incompatibilities were found within the RH/KEL system. A total of 752 packed RBC units were transfused during a pregnancy or immediately after a delivery, representing 11% of the transfused packed RBC units. Specifically, 478 packed RBC units (7%) were transfused for pregnancy monitoring, and 274 packed RBC units (4%) were used to treat sickle cell complications during pregnancy. There were 544 packed RBC units that were transfused outside of French Guiana, and no information was found regarding the site of the transfusion for 361 participants.

### Characteristics of the Alloimmunized Patients

Taking into consideration only the transfused patients (*n* = 298), 68 (23%) were alloimmunized. We did not take in account the alloimmunization rate, because the study design not allowed calculating incidences.

### The Hemoglobin Phenotypes

No significant difference in the hemoglobin phenotypes was found between the alloimmunized and the non-immune patients. Molecular analysis of variant antigens and rare phenotypes was performed in 37 (54%) alloimmunized patients. For the alloimmunized patients who had undergone few transfusions (<30 packed RBC units), this molecular biology analysis was conducted in 28 patients (51%). Comparison of the alloimmunized vs. the non-alloimmunized patients revealed that the alloimmunized patients had undergone significantly more transfusions (*p* = 0.02), with more packed RBC units (*p* = 0.01) and in emergency settings (*p* = 0.01), as simple transfusions (as opposed to exchange transfusions). Twenty-five percent of the alloimmunized patients were in the exchange transfusion program vs. 18% of the non-alloimmunized patients, although this difference was not statistically significant. In multivariate logistic regression, only the mean number of single transfusions was statistically higher in the alloimmunized patients (*p* < 0.04).

### RBC Antibodies

The 68 alloimmunized patients produced a total of 134 RBC alloantibody specificities. Table 1 shows the specificities of the antibodies that were identified, with 42 (31%) belonging to the Lewis blood group system, of which 22 (52%) had anti-Le^a^ specificity. The MNS and Rh were the next most frequent blood group systems involved, with 28 (21%) involving MNS and 25 (19%) involving the Rh system. The Kidd system accounted for 12 (9.0%) of the alloantibodies. The Kell and Duffy systems accounted for 8 (6.0%) alloantibodies each, and the Lutheran system for 3 (2%). Twenty-one transfused patients (7%) had at least one alloantibody that was known to be dangerous, namely anti-Jkb (JK2), anti-S (MNS3), and anti-Fya (FY1). Of the 68 alloimmunized patients, 10 (15%) had at least one associated autoantibody. One of the patients with multiple (three) antibodies had an antibody that we were unable to identify.

### Characteristics of the Patients Who Developed a Delayed Hemolytic Transfusion Reaction

Thirteen (19%) of the patients with alloimmunization developed a DHTR, thus representing 4.4% of all of the transfused patients. The average age at the time of the DHTR was 29 (12–52) years. There were nine women. Ten of these patients were homozygous SS and three were SC. The SC patients were exclusively pregnant women. Among patients with DHTR, 11 were antibody positive and two antibody negative. Six of them had antibodies known to be dangerous. The specificities of these alloantibodies are detailed in Table 2.

**Table 2:** Specificities of RBC alloantibodies identified in the 13 patients with DHTR.

**RBC Alloantibodies specificity**	**Number**
Anti FY1 (Fya)	4
Anti FY2 (Fyb)	1
Anti JK1 (Jka)	2
Anti JK2 (Jkb)	3
Anti LE1 (Lea)	4
Anti LE2 (Leb)	2
Anti LE3 (Lea+b)	2
Anti MNS1 (M)	1
Anti MNS2 (N)	0
Anti MNS3 (S)	2
Anti MNS30	1
Anti RH1 (D)	1
Anti RH2 (C)	2
Anti RH3 (E)	2
Anti KEL1 (K)	1
Anti KEL3 (Kpa)	2
Anti LU1 (Lua)	1
Anti DI3	1
Anti Dumbrock 2	1
Non-specific	1

## Discussion

### Epidemiological Characteristics

Our study population was similar to other populations of sickle cell patients, with the usual predominance of young sickle cell patients and the homozygous SS phenotype as the most represented. There was a notably higher proportion of the SC phenotype compared to the proportion found in mainland France, the West Indies, and in South and Central America (13, 14). This proportion was, however, roughly the same as the proportion reported for Guadeloupe (15), and the higher frequency of SC SCD in the French West Indies and Guiana could be due to a different ethnicity of the populations (hemoglobin C is more common in West Africa, especially in Ghana) (16, 17). The characteristics of the sickle cell population in terms of the RH/KEL phenotype were comparable to those that have been described in other populations (18, 19). The distribution of ABO blood groups was the same as that observed in sickle cell patients monitored at the Henri-Mondor Hospital (Créteil, France); the most frequent Rh phenotype in sickle cell patients being D+ C- E- c+ e+ K- (18–20). There was limited expression of the Fya, Jkb, and S antigens in our population. These antigens have been reported to be expressed much more in the Caucasian population (18, 19). In our study, 60% of the sickle cell patients had been transfused. This proportion is identical to the proportion reported in Guadeloupe (15), although the proportion reported in the literature is often higher (21, 22). Chou et al. reported that nearly 90% of all adult sickle cell patients had been transfused at least once (21). The proportion of transfused adult sickle cell patients in the current study is reportedly 77%, which is lower than the rates in the study by Chou. The current study could have underestimated the number of patients receiving transfusions (e.g., lack of centralized reporting system as some hospitals have computer records and some have paper records). Or, perhaps the rates are lower due to the efficacy of therapeutic intensification with hydroxyurea, the high proportion of SC phenotypes in our study or a loss of information regarding transfusions for patients who were transfused outside of French Guiana. The number of transfused packed RBC units increased over time. There are several possible reasons for this increasing trend in blood transfusions among SCD patients. Firstly, with improvements in transfusion medicine, clinicians may be treating the acute complications of SCD more aggressively, with a more frequent use of exchange transfusions that require more blood (23). Secondly, the number of packed RBC units per transfusion is expected to increase with age and weight. There is no national database for transfused patients, and the regional nature of the current database implies a loss of data when patients move from one region to another. The same is true for transfusions performed outside of France. For some of the packed RBC transfusions, no information was available as to where these transfusions were performed. Group O packed RBCs accounted for a large proportion (65%) of the total units used, and this was 12% higher than the proportion of O blood group patients in our study population. This could be due to the particular transfusional logistics in French Guiana, which involves frequent use of group O packed RBCs. An evaluation carried out by the French Blood Establishment in 2016 also described overuse of Group O packed RBCs. Emergency distribution from the blood bank supplies of the Western French Guiana Hospital may underlie this phenomenon (unpublished data). Indeed, O-phenotype packed RBCs were used in nearly 20% of cases, which supports this hypothesis. It should be noted that the low frequency of the D+ C- E- phenotype in Caucasian donors leads to a frequent diversion of RBC phenotype D-, which are themselves not readily available because of their storage and use in emergency blood banks. For example, in Île-de-France, in nearly 20% of cases, sickle cell patients with the D+ C- E- phenotype receive D- C- E- packed RBCs (18, 24). According to the French Blood Establishment, there do not appear to be any problems with the supply of labile blood products in French Guiana, other than the additional time required for their delivery. On the other hand, the origin of the packed RBCs can vary according to the immunological needs, which allows optimized phenotypic adequacy. The need for packed RBCs is steadily increasing due to the increasing number of people being monitored, as well as the increase in individual needs per patient through the establishment of long-term transfusion programs. It is recommended that poorly preserved packed RBCs are transferred in these patients who are dependent on their transfusion support, within 15 days as a general rule. “Fresh” RBCs allow for better transfusion efficiency and thus an increase in the time between transfusions. In addition, a study in mice has shown an impact of RBC age on alloimmunization (23, 25).

### High Prevalence of Alloimmunization

In our study, RBC alloimmunization occurred in 23% of the transfused SCD patients. This alloimmunization rate increased and the number of alloantibodies and transfused units increased with time. This prevalence would have been even higher if our study population did not exclusively received transfusions with blood systematically matched for C, c, E, e, and K antigens. This prevalence could have been lower if the search for antigenic variants by molecular biology had been carried out in all of the transfused patients. The rate of alloimmunization reported in the literature varies between studies from 4 to 47%, with a median of 25% (18, 26, 27). A meta-analysis has suggested a rate of alloimmunization ranging from 4.4 to 76% (28). Our prevalence was higher than in Guadeloupe (15). The prevalence of alloimmunization at the Henri-Mondor Hospital in Créteil (France) is higher, and this may well be due to the fact that this “reference center” monitors patients who require complex disease management (18). In Brazil, the rate of alloimmunization has been reported to be as low as 9.9% (29). The authors suggest that high rates of alloimmunization are due to the disparity of antigenic frequencies between blood donors of mostly Caucasian origin and the SCD recipients, who are more often Afro-Caribbean. This explains a lower rate of alloimmunization in countries where the donors and the recipients are of the same ethnic origin (19). In the United States of America, alloimmunization occurs in 30% of all transfused sickle cell patients, compared to only 2–5% for other non-sickle cell transfusion recipients. This may be explained by the immunocompetence of sickle cell patients compared with other transfused patients whose underlying pathologies often induce immunosuppression (30). According to Chou et al., alloimmunization affects 18–76% of sickle cell patients transfused with packed RBCs compatibilized based on the ABO and Rhesus systems, vs. 5–14.5% when the packed RBCs are compatibilized based on the ABO Rhesus D, C, E, and K system (23). Several other studies support this hypothesis, stating that extensive compatibility with the Jkb, Fya, and S antigens would further reduce the risk of alloimmunization (31, 32). RBC alloimmunization occurs due to genetic disparity between donor and recipient antigens. Indeed, the most frequent phenotype in sickle cell patients is D+ C- E-c+ e+ K-. Thus, the latter is present in 50 to 75% of individuals of Afro-Caribbean origin, while its frequency is <2% in individuals of European origin (18, 20). Sickle cell patients in Uganda and Jamaica, where donors and recipients tend to be from the same ethnic population, have lower alloimmunization rates of 6.1 and 2.6%, respectively. It should be noted, however, that the low use of transfusion in these countries in the management of SCD (due to reduced transfusion safety and product availability) also contributes to these lower rates of alloimmunization (19, 33), as highlighted in a study comparing Jamaica to the United Kingdom, where this rate was found to be 76% (34). We are inclined to presume that the rate of alloimmunization was lower when blood donations were available in French Guiana before 2006. However, we have no collected data to support this hypothesis, and this is one of the limitations of our study. A study published in 2015 showed a prevalence of 16% (35). Reliance on intra-ethnic transfusion might decrease the rate of alloimmunization. Some studies, however, suggest that intra-ethnic transfusions may not completely prevent the risk of alloimmunization, due to the existence of antigenic variants found in populations of African origin (19, 27). In our study, the most frequently encountered antibodies were anti-LE1, anti-MNS1, and anti-LE2, thus confirming the results published in 2015 (35). In a recent study, Allali et al. (36) had also reported a high level of these alloantibodies. Antibodies from the Rh system were found less often. The frequency of antibody types found in our study also appears to be comparable to that described in the United Kingdom (34). In the literature, the antibodies are mainly of the Rh system (especially anti-RH2 and anti-RH3 antibodies) and the Kell system (26, 28). This is the case for Guadeloupe, where the predominant antibodies are anti-RH2 and anti-RH3 (15). In addition to these two antibodies, anti-RH1 is also frequently found in the United States and Brazil (27, 29, 37). In our study, we noted the presence of natural antibodies. Indeed, these antibodies are said to be irregular when they are observed sporadically in the serum of individuals whose RBCs do not express the corresponding antigen. This is the case of antibodies directed against antigens of the Lewis system (26, 38). In addition, some authors claim that anti-Lewis alloimmunization can sometimes occur during pregnancy, due to a loss of expression of the Lewis system. The mechanism remains unknown at present. A bacterial origin or a placental interaction have been hypothesized (38). The main reported risk factors for RBC alloimmunization in SCD patients include age, age at the time of the first transfusion, the number of RBC units received, autoantibody formation, increased inflammation during transfusion, and the age of the RBC units (39, 40). In our study, no significant difference was found between alloimmunized and non-immune patients in terms of their average age, gender, or the hemoglobin phenotype. The data in the literature on this subject are contradictory (26). A number of studies have, however, also not found differences between alloimmunized and non-immunized patients (26, 33). Other authors suggested that alloimmunization occurs more often in women, which may be partly explained by exposure during pregnancy for some, or independently of pregnancy for others. This alloimmunization increases with age and is independent of the number of transfusions (28, 29, 41). In a study by Murao et al., alloimmunization occurred more frequently in sickle cell patients with the SC phenotype (29). In our study, the average age of the patients with alloimmunization was 20 years, and they received an average of 7.15 packed RBC units, which is consistent with the data in literature (33, 37). Multiple studies have found that the risk of alloimmunization appeared early in the transfusion history and increased with the increase in the number of transfusions (2, 19, 28, 29). We also found this difference in the total number of transfusions in univariate analysis, which was not significant in multivariate analysis. In addition, there was a significant difference in the number of emergency transfusions as well as in episodic transfusions. Only the number of episodic transfusions was found to be significantly different in multivariate analysis. This corroborates the results of a meta-analysis that similarly found a risk of increased alloimmunization with episodic transfusions (28).

### Delayed Hemolytic Transfusion Reaction

One complication of alloimmunization is delayed hemolytic transfusion reaction/hyperhemolysis (DHTR/H) syndrome. In our study, 4.4% of the patients developed hyperhemolysis. This result is in keeping with the data in the literature (7, 42). According to several authors, the occurrence of DHTR/H is underdiagnosed as this complication is still poorly understood and it can go unnoticed (19, 42). Indeed, there is no item dedicated to DHTR in the declaration of adverse reactive effects published by the French National Agency for the Safety of Medicinal Products. DHTR are, therefore, declared in other items, such as “Sickle cell hemolysis,” “Hemolysis other,” “Immunological incompatibility,” “Transfusion inefficiency,” “Unlisted/unspecified diagnoses.” In addition, there is no coding of the underlying pathology.

### Limitations

Other limitations of our study include information bias on the notion of episodically or chronically transfused patients, for patients transfused outside of French Guiana. Although it is highly likely that the phenotypes of the RBC of individuals from French Guiana (compared with blood from France) are more similar to those of transfused sickle cell patients, we could not collect data to demonstrate this. However, while the study shows that alloimmunization rates are increasing, and the blood is from other territories in France, the increased rates could be due to increased number of transfused units, not the increase in the number of transfused patients. Despite these limitations, this comprehensive cross-sectional study has demonstrated increased alloimmunization rates in a sickle cell population that is totally dependent on blood from donors in other territories of France.

## Conclusions

This cross-sectional study was designed to determine the RBC alloimmunization rate in patients with SCD in French Guiana since the transition away from blood donation in French Guiana. The study reports a 23% alloimmunization rate, in which both the number of alloantibodies and transfused RBC units increases over time (years). In order to reduce this high rate of alloimmunization, since 2015, the French Blood Establishment has devised careful transfusion strategies for patients with RBC alloantibodies. However, the reestablishment of blood donation in French Guiana could help reduce this high prevalence of alloimmunization.

## Data Availability Statement

The raw data supporting the conclusions of this article will be made available by the authors, without undue reservation.

## Author Contributions

SC and NE analyzed the data and drafted the manuscript. VV, MP, PS, and FM collected the data, provided the necessary logistical support, and read the manuscript. NE and FM provided critical comments on the manuscript. All authors contributed to the article and approved the submitted version.

## Conflict of Interest

The authors declare that the research was conducted in the absence of any commercial or financial relationships that could be construed as a potential conflict of interest.
